# Regional Dietary Pattern Associated with the Risk of Hypertensive Dyslipidemia Multimorbidity in Chinese Elderly: Results from China Nutrition and Health Surveillance in 2015–2017

**DOI:** 10.3390/nu17050852

**Published:** 2025-02-28

**Authors:** Jiaxi Li, Liyun Zhao, Dongmei Yu, Xingxing Wu, Hongyun Fang, Weiyi Gong, Pengfei Wang, Lei Hua, Lahong Ju

**Affiliations:** 1 National Institute for Nutrition and Health, Chinese Center for Disease Control and Prevention, Beijing 100050, China; jiaxijy6@163.com (J.L.);; 2 NHC Key Laboratory of Public Nutrition and Health, Chinese Center for Disease Control and Prevention, Beijing 100050, China

**Keywords:** elderly, regional dietary patterns, hypertensive dyslipidemia multimorbidity

## Abstract

**Objectives:** This study investigated regional dietary patterns and their association with hypertensive dyslipidemia multimorbidity among older Chinese adults. **Methods:** Data from 13,809 individuals (aged ≥ 65 years) were extracted from the 2015–2017 China Nutrition and Health Surveillance. Hypertensive dyslipidemia multimorbidity was defined as concurrent hypertension and dyslipidemia. Four dietary patterns were identified via exploratory factor analysis using 3-day 24 h dietary records. **Results:** Four dietary patterns were extracted: traditional southern, diverse, high oil and salt, and animal oil–other animal meat–coarse grain pattern. The national prevalence of hypertensive dyslipidemia multimorbidity was 31.9%, with significant regional variation (highest in North China: 36.4%; lowest in Central China: 27.9%; *p* < 0.0001). Regional dietary dominance included: the traditional southern pattern in South China (75.9%); the traditional southern (35.8%) and diversified (28.8%) patterns in East China; animal oil–other animal meat–coarse grain (38.4%) and traditional southern (37.9%) patterns in Southwest China; high oil and salt (36.8%) and traditional southern (25.9%) in Central China; diversified (47.7%) and high oil and salt (26.3%) patterns in North China; high oil and salt (44.9%) and diversified (37.9%) patterns in Northwest China; and the diversified (46.9%) pattern in Northeast China. In the highest scoring group of the animal oil–other animal meat–coarse grain pattern, the risk of hypertensive dyslipidemia multimorbidity was 1.179 times greater compared with the lowest scoring group (Q4 vs. Q1, OR = 1.179, 95% CI: 1.032–1.316, *p* < 0.001). Region-specific analyses highlighted: increased risks with the traditional southern pattern in South/East China (Q4 vs. Q1, OR = 2.080, 95% CI: 1.036–4.175, *p* < 0.001) and (Q4 vs. Q1, OR = 1.734, 95% CI: 1.052–2.859, *p* < 0.001); protective effects of the diversified pattern in Northwest China (Q4 vs. Q1, OR = 0.377, 95% CI: 0.173–0.824, *p* < 0.001); elevated risks with the high oil and salt pattern in Central/North China (Q4 vs. Q1, OR = 2.343, 95% CI: 1.107-4.955, *p* < 0.001) and (Q4 vs. Q1, OR = 1.837, 95% CI: 1.019–3.312, *p* < 0.001); and higher risks with the animal oil–other animal meat–coarse grain pattern in Southwest China (Q4 vs. Q1, OR = 2.009, 95% CI: 1.004–4.021, *p* < 0.001). **Conclusions:** In conclusion, dietary patterns among elderly Chinese are diversified and should be optimized based on local food resources to control hypertensive dyslipidemia multimorbidity.

## 1. Introduction

As one of the major public health problems in the world, chronic non-communicable diseases (NCDSs) are characterized by hidden onset and long course of disease, which bring many challenges to patients’ health, family burden, and even social impact [1]. Complex etiological mechanisms result in the interaction between many chronic diseases, resulting in individuals possibly suffering from multiple chronic diseases at the same time [2,3,4,5,6,7]. The phenomenon of multiple diseases is common in the elderly population, which seriously threatens the physical and mental health and quality of life of the elderly population [8,9,10]. According to the 2020 Report on Nutrition and Chronic Diseases of Chinese Residents, hypertension and dyslipidemia are the most common chronic diseases in Chinese aged 60 and above, with prevalence rates of 59.2% and 36.4%, respectively [11]. Studies have shown that up to 61.5% of hypertensive patients have dyslipidemia [12]. Another study showed that 65.8% of patients with dyslipidemia had high blood pressure [13]. It is suggested that hypertension and dyslipidemia have an obvious aggregation tendency. Both hypertension and dyslipidemia are independent risk factors for cardiovascular diseases [14]. The results of a large-sample population genetic epidemiological study published in 2019 suggest that the exposure duration of an individual with elevated blood pressure and lipid levels is positively correlated with the risk of cardiovascular diseases, and the two have a synergistic effect in the course of cardiovascular diseases [15]. When individuals suffer from both hypertension and dyslipidemia, the risk of cardiovascular and cerebrovascular diseases is multiplied [16]. Hypertensive dyslipidemia multimorbidity is one of the common chronic disease comorbidities in hospitalized patients in the United Kingdom [17], and with a prevalence of 39.2% in people aged 65 years and older in the United States, according to the 2013 US HCUP NIS data. Data from 2012 to 2016 U.S. clinical studies show that the prevalence of hypertensive dyslipidemia multimorbidity among adults 65 years and older in Florida is as high as 49.9% [18]. Yaoda [19] conducted a meta-analysis on the prevalence of chronic diseases in China during 2002–2022 and found that the prevalence of hypertensive dyslipidemia multimorbidity in elderly people over 60 years old was 13.1%. According to the monitoring data of chronic disease and risk factor monitoring of China in 2018, the prevalence rate of elderly people aged 65 to 75 was as high as 27.7% [20]. At present, the prevalence of hypertension and dyslipidemia in the elderly population in China is at a high level.

In addition to genetic factors and age, unreasonable diet is a risk factor leading to the accumulation of hypertension and dyslipidemia in individuals [21,22,23]. Studies have shown that excessive intake of salt can lead to sodium and water retention and increase blood pressure [24]. Excessive consumption of red meat, which is rich in saturated fatty acids, can raise the level of LDL cholesterol in the blood [25]. Excessive intake of alcohol and carbohydrates can also lead to increased lipid concentrations in the blood [26,27]. In the 30 years since 1990, the diet of Chinese residents has undergone great changes, with a significant increase in red meat intake and a significant decrease in grain and vegetable intake [28]. A study in *The Lancet* on diet and health in 195 countries pointed out that Chinese residents have the highest intake of salt in cooking and most insufficient intake of multi-grains/fruit in the world [29]. The threat to the health of the Chinese population is even more serious.

There are obvious regional differences in the dietary habits and food consumption of residents in different regions of China. Geographical location, food culture, market accessibility, and food supply institutions will have a significant impact on the dietary behaviors of residents in different regions [30,31]. Some studies have found that the dietary patterns of elderly people in China vary from south to north [32]. Qin Erdai [33] and Ma Zhimin [34] found through a literature review that the dietary patterns of residents in different regions present differences, and the risk of chronic diseases is also different in different regions. Some foreign studies have also reported the spatial distribution characteristics of dietary patterns and suggested that the study of regional dietary patterns is of great significance to improve the effectiveness and compliance of dietary intervention [35,36,37]. At present, there are few studies on the association between regional dietary patterns and chronic disease comorbidities. Therefore, this study used the data from the China Nutrition and Health Surveillance 2015–2017 to explore whether regional dietary patterns are associated with the prevalence of hypertensive dyslipidemia multimorbidity in Chinese elderly people and to provide data support for developing accurate and effective regional dietary improvement measures tailored for Chinese elderly people, so as to reduce the incidence of hypertensive dyslipidemia multimorbidity among them.

## 2. Materials and Methods

### 2.1. Data Sources

The data in this study were collected from the Chinese adult chronic disease and nutrition monitoring population aged 65 years and above from 2015 to 2017. A stratified and multi-stage random sampling method was used to analyze the representative samples of 298 monitoring sites in 31 provinces (autonomous regions/municipalities directly under the central government). The study included the following indicators: (1) age 65 years and above; (2) study subjects who have completed personal basic information surveys, physical examinations, laboratory tests, and dietary surveys; and (3) individual energy intake: the average energy intake was calculated based on a 24 h dietary survey for 3 days, and the energy range was 500–7000 kcal. A total of 13,809 participants were included. All subjects signed informed consent prior to the investigation, and this study has been supported by the Ethics Committee of the Chinese Center for Disease Control and Prevention (approval number: 201519-B).

### 2.2. Basic Information Survey

Basic information (sex, age, urban and rural area, geographical location, marital status, educational level, BMI, smoking status, alcohol consumption, family history of hypertension, physical activity level, per capita household income, sleep duration, and sedentary duration) was collected face-to-face from all families and individuals.

### 2.3. Dietary Information Survey

The dietary survey was conducted by using a 24 h dietary review method and weighing record method for 3 consecutive days. The 24 h dietary intake of the surveyed subjects for 3 consecutive days (2 working days, 1 rest day) was collected. Meanwhile, the consumption of oil and salt and other major condiments in the household for 3 consecutive days was recorded with food weighing, and the number of family diners for 3 days was recorded [31,38,39]. The collected food was divided into 21 kinds of food: rice and rice products, pork, fresh vegetable, seafood, poultry, whole grain, wheat and wheat products, fresh fruit, milk, eggs, Chinese pastry, nut, soya bean, vegetable oil, cooking salt, animal oil, other animal meat, potato, mixed beans, sugar and confectionery, animal viscera.

### 2.4. Physical Examination

In order to reduce measurement bias, instruments of the same model and brand were used to measure all items in this study. Height was measured using a TZG altimeter from Wuxi Weighing Apparatus Factory Co., Ltd., located in Wuxi, Jiangsu Province China. The weight was measured using an electronic weight scale, model TANITA HD-390, manufactured by Dongguan Bailida Health Equipment Co., Ltd., based in Dongguan, Guangdong Province, China. The blood pressure was measured using an electronic blood pressure monitor, model Omron HBP1300, from Omron Dalian Co., Ltd., Dalian, Liaoning Province, China. These instruments have an accuracy of 0.1 cm, 0.1 kg, and 1 mmHg, respectively.

### 2.5. Laboratory Testing

A total of 8 mL of fasting blood was collected at one time to determine fasting blood glucose, total cholesterol, triglyceride, LDL-C, HDL-C, blood uric acid, and hemoglobin. The above measurements were carried out by professionals in the laboratory with strict quality control.

### 2.6. Definition of Hypertensive Dyslipidemia Multimorbidity

The subjects suffered from both hypertension and dyslipidemia. The criteria for the determination of hypertension were: patients diagnosed with hypertension with systolic blood pressure ≥ 140 mmHg and/or diastolic blood pressure ≥ 90 mmHg, as determined by on-site blood pressure measurement [21]. The criteria for determining dyslipidemia were as follows: abnormal TC ≥ 6.2 mmol/L, LDL-C ≥ 4.1 mmol/L, HDL-C < 1.0 mmol/L, TG ≥ 2.3 mmol/L can be determined as dyslipidemia by laboratory testing [4].

### 2.7. Covariates

The types of variables used for multiple adjustments in logistic regression analysis were defined as follows: (1) Age was divided into general elderly (65~79 years old) and senior elderly (>80 years old). (2) Sex was divided into male and female. (3) Residential zoning was divided into urban and rural areas. (4) Geographical location was divided according to the administrative divisions and geographical divisions of China. North China includes Beijing, Tianjin, Hebei, Shanxi, and Inner Mongolia Autonomous Region; Northeast China includes the Heilongjiang, Jilin, and Liaoning provinces; East China includes the Shanghai, Jiangsu, Zhejiang, Anhui, Jiangxi, Shandong, and Fujian provinces; Central China includes the Henan, Hubei, and Hunan provinces; South China includes Guangdong Province, the Guangxi Zhuang Autonomous Region, and Hainan Province; Southwest China includes Chongqing Municipality, Sichuan Province, Guizhou Province, Yunnan Province, and Tibet Autonomous Region; and Northwest China includes Shaanxi Province, Gansu Province, Qinghai Province, Ningxia Hui Autonomous Region, and Xinjiang Uygur Autonomous Region. (5) Marital status was classified as married/cohabiting and unmarried/separated/divorced/widowed. (6) Education level was divided into primary school and below, junior high school, senior high school, and above. (7) The body mass index (BMI) was classified according to the Chinese Obesity Working Group as underweight (BMI < 18.5), normal (18.5 ≤ BMI < 24), and overweight/obese (BMI ≥ 24) [40]. (8) Smoking status was divided into smoking (always smoked, ever smoked), passive smoking (second-hand smoke), and never smoked. (9) Drinking status was divided into drinking and non-drinking. (10) Family history of hypertension was divided according to whether parents, grandparents, maternal grandparents, and siblings suffered from hypertension. (11) Physical activity status was determined based on the calculation method of physical activity levels in the International Physical Activity Questionnaire (IPAQ). This classification was based on the weekly total metabolic equivalent of task (MET) and the total weekly duration of physical activity. Participants were categorized into three levels: low (MET < 600), moderate (600 ≤ MET ≤ 3000), and high (MET > 3000) [41]. (12) According to the per capita household, income was divided into low (<10,000 CNY/year), medium (10,000 to 20,000 CNY/year), high (>20,000 CNY/year), and missing. (13) Sleep status was divided into less than 7 h/day, 7–9 h/day, and more than 9 h/day. (14) Sedentary time was divided into less than 2 h/day, 2 to 4 h/day, and more than 4 h/day.

### 2.8. Recommended Dietary Guidelines

The *Dietary Guidelines for Chinese Residents* (2022) [24] recommends that each person aged ≥ 65 years should consume 200–300 g of cereals per day, including 50–150 g of whole grains and beans, and 50–100 g of potatoes; 300~500 g of vegetables and 200~350 g of fruit; 120~200 g total intake of fish, poultry, meat, eggs, and other animal food, including 40~75 g of livestock and poultry meat, 40~75 g of fish, shrimp, crabs, and shellfish, and 50 g of egg; and 25~35 g of soy and nuts and 300~500 g of milk and dairy products. The cooking salt recommendation is less than 5 g, and cooking oil usage should not be more than 25~30 g.

### 2.9. Dietary Patterns

According to the food and weighing records of cooking oil and cooking salt in the 3-day 24 h dietary database, 21 food groups were identified. In this study, an exploratory factor analysis (EFA) was used to identify dietary patterns. The suitability of factor analysis was assessed using the Kaiser–Meyer–Olkin (KMO) test (KMO > 0.6) and Bartlett’s test of sphericity (*p* < 0.05). A principal component analysis (PCA) was employed for model estimation, and factor extraction was conducted using the VARIMAX rotation method. The number of common factors was determined based on eigenvalues (>1.05), the scree plot, and expert knowledge. Each model is named for a food variable feature with an absolute factor load of greater than 0.3. The representative dietary pattern of each survey subject was the pattern with the highest score. Subjects were divided into four groups according to the quartile of the dietary pattern score, and the daily intakes of major food components under each dietary pattern were described.

### 2.10. Statistical Analysis

All the statistical analysis was completed by sas9.4, and the figures were created using R 4.4.1. Continuous variables are described in terms of the mean and standard deviation, and categorical variables are described in terms of the count and component ratio. The description of disease prevalence and 95% CI was weighted using the PROC SURVEYFREQ program in SAS. The sample weights were based on 2010 data from the National Bureau of Statistics of China, and the chi-square was used to test statistical differences in categorical variables among groups. A factor analysis was performed using PROC FACTOR in SAS. The scores of different dietary patterns were divided into four groups according to the quartile method, and the lowest group was taken as the reference group. Multiple logistic regression was used to analyze the odds ratio (OR) and 95% confidence interval (95% CI) between dietary patterns and hypertension and dyslipidemia, and all covariables were converted into dummy variables. Model 1 was a crude model with no adjusted covariates, Model 2 was adjusted for age and sex, and Model 3 was further adjusted for BMI, physical activity, education, smoking status, marital status, household per capita income, geographical location, alcohol consumption, sleep duration, sedentary time, and family history of hypertension. *p* < 0.05.

## 3. Results

### 3.1. Characteristics of Participants

The characteristics of 13,809 elderly people are shown in Table 1. There were significant differences in sex, age, urban and rural areas, geographical location, marital status, educational level, BMI, smoking status, drinking status, family history of hypertension, physical activity level, per capita family income, sleep duration, and sedentary duration among Chinese elderly aged 65 and above (*p* < 0.01).

### 3.2. Dietary Patterns of the Elderly in China

Four dietary patterns were extracted through a factor analysis. The KMO value was 0.65, and Bartlett’s test of sphericity yielded a *p*-value of <0.001. A total of four dietary patterns were defined to explain 29.72% of the variance of 21 food variables. Table 2 shows the factor load of each food group under the four dietary patterns. Pattern one was named the “traditional southern diet model”, pattern two was named “diversified diet model”, pattern three was named “heavy oil and heavy salt model”, and pattern four was named “animal oil–other animal meat–coarse grain model”.

### 3.3. Characteristics of Food Intake in Chinese Elderly Under Different Dietary Patterns

Table 3 presents the average daily intake of various food categories among older adults under different dietary patterns. Compared with other dietary patterns, the intake of rice and rice products, fresh vegetables, seafood, and pork was higher in the traditional southern dietary pattern, and the intake of whole grains, wheat, and wheat products was lower. The elderly population following a diversified dietary pattern tends to have higher intakes of fresh vegetables, fish and shrimp, fresh fruits, dairy products, eggs, Chinese pastries, and nuts. The intake of vegetable oil and cooking salt was higher in the elderly under the Heavy oil and heavy salt dietary mode. The intake of animal oil, other animal meat, potato, and Mixed beans was greater in old people in the animal oil–other animal meat–coarse grain pattern, and vegetable oil intake was less.

### 3.4. Distribution of Four Dietary Patterns Among the Elderly in Different Regions of China

**Figure 1:**
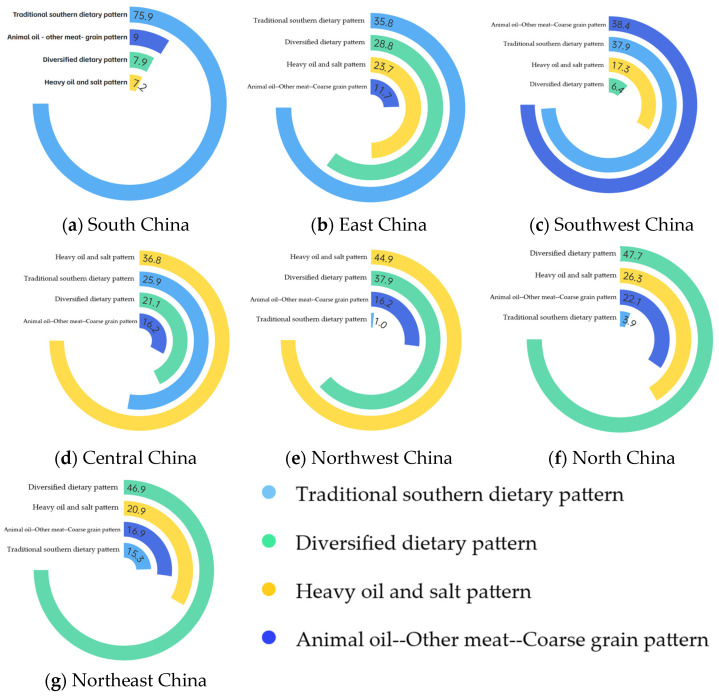
Proportions of four dietary patterns for the elderly in different regions of China: (**a**) South China; (**b**) East China; (**c**) Southwest China; (**d**) Central China; (**e**) Northwest China; (**f**) North China; (**g**) Northeast China.

The distribution of regional dietary patterns among the elderly in China is shown in Figure 1. For more information on the proportion of dietary patterns in each region, please refer to Supplementary Table S1. The elderly in South China mainly adopted a traditional southern diet pattern, accounting for 75.9%; in East China, the elderly mainly adopted a traditional southern diet pattern and diversified diet pattern, accounting for 35.8% and 28.8%, respectively. In Southwest China, the elderly mainly adopted an animal oil–other animal meat–coarse grain diet pattern and traditional southern diet pattern, accounting for 38.4% and 37.9%, respectively. In Central China, the heavy oil and salt diet pattern and traditional southern diet pattern were dominant, accounting for 36.8% and 25.9%, respectively. In North China, the elderly mainly adopted the diversified diet pattern and Heavy oil and salt diet pattern, accounting for 47.7% and 26.3%, respectively. In Northwest China, the Heavy oil and heavy salt diet pattern and diversified diet pattern were the main dietary patterns, accounting for 44.9% and 37.9%, respectively. In Northeast China, the elderly mainly adopted diversified dietary patterns, accounting for 46.9%.

### 3.5. Weighted Prevalence of Hypertensive Dyslipidemia Multimorbidity in the Elderly

The weighted prevalence of hypertensive dyslipidemia multimorbidity in the elderly over 65 years of age in China was 31.9% (95% CI: 30.6–33.2%). The weighted prevalence of hypertensive dyslipidemia multimorbidity in the elderly was the highest in North China (36.4%) (95% CI: 33.3–39.4%), and the lowest prevalence was in Central China (27.9%) (95% CI: 25.5–33.5%). There were statistically significant differences in the weighted prevalence of hypertensive dyslipidemia multimorbidity in the seven regions (*p* < 0.001). The prevalence of hypertension dyslipidemia comorbidities in Chinese elderly aged 65 and above with different characteristics is shown in Table 4.

### 3.6. Analysis of the Association Between Dietary Patterns and Comorbid Hypertension Dyslipidemia in Elderly Chinese Individuals

Table 5 presents the results of the logistic regression analysis. After multiple model adjustments, the animal oil–other animal meat–coarse grain pattern showed a positive association with hypertensive dyslipidemia multimorbidity. The highest quartile of this pattern was associated with a 1.179-fold increased risk compared to the lowest quartile (Q4 vs. Q1, OR = 1.179, 95% CI: 1.032–1.316, *p* < 0.001). No statistically significant associations were observed between hypertensive dyslipidemia multimorbidity and the traditional southern, diverse, or high oil and salt dietary patterns (*p* > 0.05).

### 3.7. Analysis of the Association Between Major Regional Dietary Patterns and Hypertensive Dyslipidemia Multimorbidity in Chinese Elderly People

The logistic regression model was used to analyze the association between common dietary patterns and hypertensive dyslipidemia multimorbidity in various regions, and the results are shown in Table 6. In South and East China, the group with the highest scores for the traditional southern dietary pattern had a risk of hypertensive dyslipidemia multimorbidity that was 2.080 times (Q4 vs. Q1, OR = 2.080, 95% CI: 1.036–4.175, *p* < 0.001) and 1.734 times (Q4 vs. Q1, OR = 1.734, 95% CI: 1.052–2.859, *p* < 0.001) higher, respectively, compared with the lowest scoring group. In Southwest China, the risk of hypertensive dyslipidemia multimorbidity was 2.009 times that of the lowest group (Q4 vs. Q1, OR = 2.009, 95% CI: 1.004~4.021, *p* < 0.001). In Central and North China, the group with the highest scores for the Heavy oil and salt dietary pattern had a risk of hypertensive dyslipidemia multimorbidity that was 2.343 times (Q4 vs. Q1, OR = 2.343, 95% CI: 1.107–4.955, *p* < 0.001) and 1.837 times (Q4 vs. Q1, OR = 1.837, 95% CI: 1.019–3.312, *p* < 0.001) higher, respectively, compared with the lowest scoring group. In Northwest China, the risk of hypertensive dyslipidemia multimorbidity was reduced in the group with the highest scores on the diversified dietary pattern (Q4 vs. Q1, OR = 0.377, 95% CI: 0.173-0.824, *p* < 0.001).

## 4. Discussion

China has a vast territory and crosses different climate zones from north to south. The output and distribution of various kinds of agricultural products are major regional factors affecting the dietary pattern of Chinese residents [42,43,44,45,46,47,48]. Through a factor analysis, this study extracted four dietary patterns of the elderly aged 65 and above in China, namely, the traditional southern dietary pattern, the diversified dietary pattern, the Heavy oil and salt dietary pattern, and the Animal oil–Other animal meat–Coarse grain dietary pattern. The elderly in different regions of China have a certain proportion of the four dietary patterns. It is suggested that the dietary patterns of the elderly in different regions of China are diversified. As China’s sustainable economic and social development creates conditions for population migration and mobility, the results of the seventh national population census in 2020 show that the floating population has increased by nearly 70% in 10 years [49]. In addition, the Internet breaks the time and space restrictions of the catering market, which is separated by time and geographical restrictions [50]; under the influence of factors such as the Internet, population mobility, geographical environment, climatic conditions, and changes in residents’ dietary habits, the dietary patterns of residents in different regions of China are no longer of a single type [39,40,49].

The distribution results of four dietary patterns of the elderly in different regions of China show that the traditional southern dietary pattern is included in the common dietary patterns in South China, East China, and Southwest China. This observation is consistent with the distribution of southern rice patterns in the south of the Qinling Mountains and Huaihe River line, which Rongping [31] found by using the Nutrition and Health monitoring data of China (2015–2017) for people over 45 years old. The “rice and pork” model obtained by Zhang Jiguo [51] using the data of the China Health and Nutrition Survey (CHNS) is also more common in the southern region. The reasons for the analysis are that the warm and humid climate in these areas is conducive to the cultivation of rice and fresh vegetables [52], rich marine resources and fishing traditions decide that local residents eat more fish and shrimp [53], and mature pig breeding technology and food culture also promote residents’ pork consumption [54]. In Northwest, North, and Central China, Heavy oil and heavy salt dietary patterns account for a large proportion of diets. Due to the relatively dry climate and the need to supplement more salt to maintain the water balance in the body, the traditional diet structure in these regions is relatively simple, and the processing of food with more cooking oil and cooking salt can increase the taste and flavor of food [55]. In addition, this may be related to the local residents liking to eat spicy food, where the preparation of spicy food is often accompanied by high oil and salt [56]. In Southwest China, the dietary pattern of animal oil, other animal meat, and coarse grain is relatively large. Southwest China is the region with the largest number of ethnic minorities, and its dietary culture is deeply influenced by the traditions of various ethnic groups. The meat of cattle and sheep not only meets the basic dietary needs but also carries profound cultural symbolic significance [57]. In addition, the complex terrain conditions in the southwest limited the extensive cultivation and supply of vegetables and fruits, resulting in a greater reliance on animal resources for diet [58]. The food composition of the diversified dietary pattern is similar to that of the Oriental dietary pattern proposed in the 2022 edition of the *Dietary Guidelines for Chinese Residents*, both of which are widely available in East China [24]. This study found that the common dietary patterns in East China, Northwest China, and North China all contain diversified dietary patterns. It is worth noting that the diversified dietary patterns are also quite common in Northwest China and North China, which may be due to the upgrading of residents’ dietary consumption and population migration, which lead to the transformation of the dietary structure of residents in North China from “grain and vegetable type” to pluralistic type. The form has changed from traditional home cooking to modern convenience [59]. Based on this trend, it can be predicted that diversified dietary patterns will occupy an increasing proportion in the future.

The findings of this study show that hypertensive dyslipidemia multimorbidity is more prevalent among females, individuals with higher educational attainment, those who are overweight or obese, passive smokers, and high-income groups. These observations align with the research findings of Yuantao [60], Xiaorong [61], and Lee [62]. Higher disease risk among females may be attributed to decreased estrogen secretion and altered secretion of other hormones following menopause in elderly women, leading to higher detection rates of hypertension and hyperlipidemia compared with males [63,64]. Studies suggest that educational attainment does not directly impact chronic disease status but indirectly instead through factors such as occupation, with higher-educated individuals typically being engaged in mental labor and demonstrating lower levels of physical activity [65]. A study in *The Lancet*, utilizing data from multiple cohorts, reported that obese individuals face approximately 12.4 times the risk of developing complex and multiple morbidities compared with those with normal weight [66]. High income is often associated with high-intensity work, excessive calorie intake, sedentary lifestyles, and poor habits such as staying up late, eating late-night snacks, smoking, and alcohol abuse. This study found no significant difference in prevalence across different levels of alcohol consumption, potentially due to survivor bias among the older study population, which does not negate the potential impact of alcohol consumption on the disease. Lee [62] used data from a ten-year cohort in South Korea to demonstrate that physical inactivity, smoking, and alcohol consumption are predictors of comorbidity among elderly Koreans.

The overall results of the logistic regression analysis indicate that, when the model was not adjusted, the lowest score group served as the reference, and there was no statistically significant difference in disease risk among the elderly population in the highest score group across various dietary patterns. Given the regional variations in morbidity among the elderly and the differing dietary patterns over time in each region, after adjusting for multiple confounding factors by region, the traditional southern dietary pattern in South and East China, the high oil and high salt dietary pattern in Central and North China, and the Animal fat–Other livestock meat–Coarse grain dietary pattern in Southwest China were positively associated with hypertensive dyslipidemia multimorbidity in elderly individuals, whereas the diversified dietary pattern in Northwest China was negatively associated. The associations between the same dietary patterns and the disease varied across different regions, potentially influenced by local factors such as climate and altitude. A study on climate and chronic diseases in China found that for every 1 °C increase in temperature, the average risk of cardiovascular diseases among residents increased by 6%, with those suffering from dyslipidemia being particularly affected by temperature changes [67]. Research by Yuting [68] also indicated a U-shaped relationship between ambient temperature and blood pressure among populations living in temperate monsoon or continental climates. Hypertension in individuals exposed to high altitudes may be related to increased hematocrit, with hypoxic erythropoiesis leading to increased blood viscosity [69]. A meta-analysis including 40,854 samples showed that for the Tibetan population, blood pressure increased by an average of 17/9.5 mmHg for every 1000 m increase in altitude [70].

In the traditional southern diet, the elderly consumed too much rice and its products in cereals, too little whole grains, too much pork, and too little soybeans, fresh vegetables, fresh fruits, milk, eggs, nuts, and other foods. The harm of pork to hypertension and dyslipidemia has been relatively clear [71,72]. Processed rice and its products have a high glycemic index, and the elderly cannot consume a large amount of energy generated rapidly due to lack of physical activity; thus, too much glucose in the blood is converted into triglycerides, and then hypertension and dyslipidemia occur [73,74,75]. Studies conducted in the Costa Rican adult population also indicated that rice intake was positively correlated with blood pressure and triglycerides and negatively correlated with HDL cholesterol [76]. Korean studies also showed that rice intake was positively correlated with triglyceride levels in blood pressure and hypertension [77,78]. Whole grains have a certain protective effect on blood total cholesterol, low-density cholesterol, and triglyceride levels [79]. The Food and Agriculture Organization/World Health Organization [80] and the Chinese Nutrition Society [81] believe that replacing refined grains with whole grains can reduce the risk of cardiovascular disease. Increasing the intake of fresh vegetables, fresh fruits, fish and shrimp, milk, eggs, soybeans, nuts, and other foods to diversify residents’ diets is conducive to the control of blood pressure and blood lipids [21,22]. Therefore, it is suggested that the elderly who mainly eat the traditional southern diet pattern can replace rice and its products with coarse grains such as brown rice, oats and Job’s tears; and reduce the intake of pork; and increase the intake of fresh vegetables, fresh fruits, fish and shrimp, soy products, milk, eggs, and nuts to ensure a balanced and diversified diet. A study in *The Lancet* on diet and health in 195 countries pointed out that the death rate of cardiovascular diseases caused by a heavy oil and salt diet in China ranked first in the world, and such a diet would also increase the risk of stomach cancer and osteoporosis, seriously damaging the quality of life of the elderly [22]. A long-term high-fat diet causes irreversible damage to the gastrointestinal tract, liver, heart, and other organs and also increases the risk of depression and anxiety in the elderly [82], seriously damaging the quality of life of the elderly. The prediction model of the Chinese population established by Monique [83] proposed that a reduction of 1 g of cooking salt intake per day by Chinese residents could prevent nearly 9 million cases of cardiovascular disease by 2030. Therefore, the elderly who mainly use the Heavy oil and heavy salt diet must control the average daily intake of cooking salt at 5 g/d and the average daily intake of cooking oil at 25 g/d~30 g/d. The average daily intake of animal oil and other animal meat was significantly higher in the elderly with the animal oil–other animal meat–coarse grain dietary pattern, although the average daily intake of whole grains, potatoes, and nuts was higher than that of other patterns, but the contribution was lower than that of animal oil and other animal meat. Animal oil and red meat such as cattle and sheep are rich in saturated fatty acids, and excessive intake will lead to an increase in blood cholesterol and low-density lipoprotein, promote the formation of arteriosclerosis, reduce the elasticity of blood vessel walls, and further cause an increase in blood pressure [84]. In the nomadic Maasai population, which relies heavily on animal products, only red meat intake was positively correlated with the prevalence of dyslipidemia, but there was no significant correlation between red meat consumption and hypertension due to the low use of cooking salt in the traditional Maasai food preparation [85]. A cross-sectional study in the United Kingdom also found that excessive intake of processed red meat was associated with higher serum total cholesterol, LDL cholesterol, and hypertension levels in women [86]. Therefore, it is recommended that elderly people with an animal oil–other animal meat–coarse grain dietary pattern reduce the intake of red meat and animal oil such as beef and mutton and increase the intake of fresh vegetables, fresh fruits, milk, eggs, and soy products to ensure a diversified and balanced diet. In this study, the diversified dietary pattern mainly consists of fresh vegetables, fish and shrimp, fresh fruits, milk, eggs, Chinese pastries, and nuts. The diversified dietary pattern is rich in food composition, which is similar to the DASH diet (DASH), which can effectively reduce blood pressure and low-density lipoprotein cholesterol [87]. The principle of DASH is to improve overall health by using a diet high in potassium, magnesium, calcium, and dietary fiber that is unsaturated fatty acid-rich and intakes saturated fatty acid in moderation [88]. Fresh vegetables and fruit in the diverse dietary pattern food group contain dietary fiber, which can bind to intestinal cholesterol and reduce blood cholesterol levels [89]. He Zhi [90] used prospective cohort data in rural China to study the association between blood pressure level and fruit and vegetable intake in hypertensive patients and found that every 100 g/d increase in total fruit and vegetable intake reduced the risk of blood pressure progression by 4%. Fish and shrimp, especially deep-sea fish, contain omega-3 fatty acids that can inhibit the synthesis of triglycerides, increase the high density lipoprotein cholesterol in the blood, reduce lipid deposition on the wall of blood vessels, repair the damaged inner wall of blood vessels, restore blood vessel elasticity, and have a positive effect on stabilizing blood pressure [91]. Studies have found that intake of omega-3 fatty acids between 2 g/day and 3 g/day can effectively reduce blood pressure [92]. Dairy products help to increase the concentration of HDL cholesterol, inhibit the oxidation of LDL cholesterol, and thus inhibit the inflammatory process [93]. At present, the daily intake of whole grains, soybeans, fresh vegetables, fresh fruits, milk, eggs, fish and shrimp, and other foods in the elderly with the diversified dietary pattern fails to meet the recommended intake in the *Dietary Guidelines for Chinese Residents* (2022 edition) [24]. Therefore, it is suggested that elderly people with diversified dietary patterns should, on the basis of the existing diet, increase the intake of whole grains, milk, eggs, fish and shrimp, soy products, nuts, and other foods to achieve diverse food composition and adequate intake.

The limitations of this study are as follows: 1. This study used 3-day 24 h dietary data of the elderly, which is specific but not representative enough in the long term. 2. The lack of family history of dyslipidemia in the elderly has a certain impact on the association analysis between dietary pattern and the prevalence of hypertension and dyslipidemia comorbidities. 3. The lack of data on the changes in basic material parameters of the food industry in different regions of China may weaken the dietary differences between different regions in China. 4. The scores of the study subjects under four dietary patterns were compared, and the pattern with the highest score was selected as their representative dietary pattern. This approach overlooked the possibility that a few study subjects might have similar scores under several patterns, thus introducing a certain degree of bias.

## 5. Conclusions

In summary, the diets of the elderly in different regions of China present diversified dietary patterns. The elderly in South China mainly adopt traditional southern dietary patterns, the elderly in East China mainly adopt the traditional southern dietary pattern and diversified dietary pattern, and the elderly in Southwest China mainly adopt the animal oil–other livestock meat–coarse grain dietary pattern and traditional southern dietary pattern. The elderly in Central China mainly adopt the Heavy oil and heavy salt dietary pattern and traditional southern dietary pattern, the elderly in North China mainly adopt the diversified dietary pattern and Heavy oil and heavy salt dietary pattern, the elderly in Northwest China mainly adopt the Heavy oil and heavy salt dietary pattern and diversified dietary pattern, and the elderly in Northeast China mainly adopt the diversified dietary pattern. The traditional southern dietary pattern, characterized by rice and pork as the main staples, is prevalent in South and East China; the high-fat, high-salt dietary pattern, dominated by excessive use of vegetable oils and cooking salt, is common in Central and North China; and the Animal oil–Other animal meat–Coarse grain dietary pattern, which is based on animal fats and meats such as beef and lamb, is prevalent in Southwest China, all showing a positive correlation with hypertensive dyslipidemia multimorbidity in the elderly population. The diversified dietary pattern dominated by fresh vegetables, fish and shrimp, fruits, milk, eggs, and nuts in Northwest China is negatively correlated with the hypertensive dyslipidemia multimorbidity in the elderly. At present, the diversified dietary pattern is the best among the four dietary patterns extracted for Chinese elderly people, so it is recommended that elderly people in various regions of China adjust their diets according to local conditions to achieve a diverse food composition and adequate intake, thereby effectively controlling the prevalence of hypertensive dyslipidemia multimorbidity in the elderly and improving their quality of life and health level.

## Figures and Tables

**Table 1:** General characteristics of elderly participants in CNHS 2015–2017.

Characteristics		Frequency	Weighted Proportion%	χ^2^	*p*-Value
age	65~79	12,239	88.2	2052.120	<0.001
	≥80	1570	11.8		
sex	Male	6989	48.1	13.884	0.001
	female	6820	51.9		
Urban and rural	Urban	6083	42.7	7.400	0.007
	rural	7726	57.3		
Regional distribution	South China	1576	16.5	86.617	<0.001
	East China	4221	33.8		
	Southwest China	2078	15.6		
	Central China	1838	8.5		
	North China	1808	7.1		
	Northwest China	1053	5.8		
	Northeast	1235	12.7		
Marital status	Separated/Solitary/Divorced/Widowed	2080	20.0	1010.498	<0.001
	Married and living with a spouse	11,729	80.0		
Education	Primary school or below	5511	40.7	156.700	<0.001
	Middle school	6270	43.3		
	High school or higher	2028	16.1		
BMI	Underweight	586	4.5	1146.700	<0.001
	Normal	5713	42.1		
	Overweight	7510	53.4		
Current smoker	Smoking	5136	36.3	54.045	<0.001
	Exposure to second-hand smoke	3656	27.4		
	No smoking	5017	36.3		
Alcohol drinking	drinking	9659	70.3	325.600	<0.001
	No drinking	4150	29.7		
Family history of hypertension	Yes	9844	71.6	317.400	<0.001
	No	3965	28.4		
Physical activity	Low	4061	29.8	118.200	<0.001
	Moderate	3727	26.9		
	High	6021	43.3		
Income	<10,000	6806	46.1	377.700	<0.001
	10,000~20,000	3025	23.5		
	≥20,000	3632	27.5		
	deficiency	346	2.9		
Sleeping time (h)	<7 h	3110	22.9	833.700	<0.001
	7~9 h	2942	20.6		
	≥9 h	7757	56.5		
Sedentary behavior (h)	0~2 h	2398	17.1	598.400	<0.001
	2~4 h	4016	29.3		
	≥4 h	7395	53.6		
Total		13,809	100.0	13.900	0.001

**Table 2:** Factor loading of food items in each dietary pattern.

Food Group	Traditional Southern Dietary Pattern	Diversified Dietary Pattern	Heavy Oil and Salt Pattern	Animal Oil–Other Animal Meat–Coarse Grain Pattern
Rice and rice products	0.825	−0.131	0.016	0.007
Pork	0.487	0.127	0.013	0.095
Fresh vegetable	0.371	0.319	0.277	0.140
Seafood	0.344	0.309	0.112	−0.215
Poultry	0.244	0.216	0.042	−0.124
Whole grain	−0.499	−0.009	0.084	0.211
Wheat and wheat products	−0.734	−0.027	0.096	0.041
Fresh fruit	−0.022	0.643	−0.011	0.148
Milk	−0.180	0.521	−0.106	0.002
Eggs	−0.188	0.483	−0.010	−0.105
Chinese pastry	−0.089	0.435	−0.059	−0.002
Nuts	0.067	0.394	0.134	0.218
Soya bean	0.074	0.176	0.207	−0.007
Vegetable oil	−0.027	−0.019	0.737	−0.354
Cooking salt	−0.002	−0.225	0.707	0.182
Animal oil	0.299	−0.246	−0.140	0.595
Other animal meat	−0.131	0.150	0.112	0.440
Potato	−0.227	−0.088	0.194	0.315
Mixed beans	0.079	0.104	0.001	0.303
Sugar and confectionery	−0.005	0.059	0.146	0.200
Animal viscera	0.140	0.051	0.085	0.179
Percentage of variance explained	0.104	0.079	0.060	0.054

**Table 3:** Average food intake of Chinese elderly with different dietary patterns, 2015–2017 (g/d).

Food Group	Traditional Southern Dietary Pattern	Diversified Dietary Pattern	Heavy Oil and Salt Pattern	Animal Oil–Other Animal Meat–Coarse Grain Pattern
Rice and rice products	251.3 ± 96.7	77.0 ± 65.8	126.6 ± 102.7	159.3 ± 112.4
Wheat and wheat products	28.2 ± 38.8	134.2 ± 93.8	129.9 ± 115.1	93.0 ± 95.0
Whole grains and\mixed Beans	4.7 ± 13.7	25.0 ± 38.5	21.6 ± 39.1	43.9 ± 72.1
Whole grain	2.3 ± 9.8	22.0 ± 37.2	19.9 ± 38.6	30.2 ± 65.2
Mixed beans	2.45 ± 9.81	3.0 ± 10.3	1.7 ± 7.6	13.7 ± 34.1
Potato	17.8 ± 37.7	30.8 ± 45.3	46.0 ± 73.9	86.2 ± 128.8
Soybeans and nuts	10.1 ± 16.9	14.8 ± 22.2	13.5 ± 24.1	13.5 ± 25.3
Soya bean	8.3 ± 15.2	9.7 ± 16.1	11.6 ± 22.2	7.9 ± 16.1
Nuts	1.8 ± 6.9	5.1 ± 14.2	1.9 ± 7.2	5.5 ± 18.8
Fresh vegetable	262.9 ± 159.5	236.1 ± 153.5	246.3 ± 158.9	251.5 ± 166.8
Fresh fruit	13.7 ± 33.2	67.6 ± 91.2	11.1 ± 31.6	24.1 ± 55.1
Milk	3.7 ± 21.7	76.8 ± 149.6	7.2 ± 31.6	9.3 ± 39.5
Eggs	11.5 ± 18.1	35.9 ± 31.8	15.0 ± 22.3	11.9 ± 19.5
Seafood	33.2 ± 55.7	20.5 ± 38.8	16.5 ± 34.7	9.1 ± 25.1
Livestock and poultry meat	86.0 ± 74.0	53.9 ± 52.9	46.1 ± 52.6	78.5 ± 76.5
Pork	68.9 ± 63.4	38.7 ± 42.4	36.2 ± 44.0	49.8 ± 56.6
Other animal meat	1.1 ± 6.2	5.4 ± 15.1	2.9 ± 11.0	18.7 ± 44.5
Poultry	14.3 ± 32.3	9.1 ± 24.0	6.0 ± 20.4	4.6 ± 16.0
Animal viscera	1.7 ± 7.8	0.8 ± 4.9	1.0 ± 6.5	5.4 ± 20.3
Cooking oil	28.2 ± 16.0	24.4 ± 15.8	57.5 ± 37.7	35.4 ± 29.0
Animal oil	3.2 ± 7.7	0.2 ± 1.6	0.7 ± 3.8	17.1 ± 25.4
Vegetable oil	25.0 ± 16.4	24.2 ± 15.8	56.8 ± 37.0	18.3 ± 18.0
Cooking salt	6.4 ± 3.0	5.8 ± 3.0	12.1 ± 5.3	9.1 ± 4.7
Sugar and confectionery	1.4 ± 4.8	2.2 ± 6.5	2.3 ± 12.3	5.2 ± 30.2
Chinese pastry	1.9 ± 8.2	13.8 ± 28.7	2.1 ± 9.7	2.6 ± 11.2

**Table 4:** Weighted prevalence of hypertensive dyslipidemia multimorbidity in Chinese elderly with different characteristics.

Characteristics		Number of Patients	Weighted Proportion% (95% CI)	χ^2^	*p*-Value
Sex	Male	1965	28.6 (26.9, 30.3)	22.251	<0.001
	Female	2401	34.9 (33.0, 36.8)		
Age	65~79	3910	32.2 (31.0, 33.5)	3.108	0.078
	≥80	456	29.1 (25.9, 32.3)		
Urban and rural	Urban	1966	31.8 (29.8, 33.7)	0.037	0.848
	rural	2400	32.0 (30.5, 33.5)		
Regional distribution	Central China	502	27.9 (25.5, 30.3)	28.028	<0.001
	North China	640	36.4 (33.3, 39.4)		
	East China	1466	34.0 (31.9, 36.0)		
	South China	478	29.0 (25.4, 32.7)		
	Northwest China	340	34.7 (30.7, 38.7)		
	Southwest China	576	28.9 (25.5, 32.3)		
	Northeast China	364	30.2 (25.7, 34.7)		
Marital status	Separated/Solitary/Divorced/Widowed	636	31.6 (28.1, 35.2)	0.027	0.870
	Married/Living with a spouse	3730	32.0 (30.6, 33.4)		
Education	Primary school or below	1545	29.3 (27.1, 31.5)	20.294	<0.001
	Middle school	2054	32.2 (30.4, 34.0)		
	High school or higher	767	37.7 (34.5, 40.9)		
BMI	Underweight	71	13.1 (8.1, 18.1)	257.545	<0.001
	Normal	1171	21.8 (20.2, 23.3)		
	Overweight	3124	41.5 (39.8, 43.1)		
Current smoker	Smoking	1565	29.4 (27.6, 31.3)	15.596	<0.001
	Exposure to second-hand smoke	1222	35.3 (32.8, 37.7)		
	No smoking	1579	31.8 (29.8, 33.8)		
Alcohol drinking	Drinking	3064	32.5 (30.9, 34.1)	2.4901	0.115
	No drinking	1302	30.5 (28.5, 32.5)		
Family history of hypertension	Yes	1286	31.2 (29.1, 33.3)	0.653	0.419
	No	3080	32.2 (30.7, 33.6)		
Physical activity	Low	1279	31.2 (28.7, 33.7)	3.890	0.143
	Moderate	1133	30.4 (28.0, 32.8)		
	High	1954	33.3 (31.5, 35.2)		
Income	<10,000	1971	28.9 (27.0, 30.8)	20.188	<0.001
	10,000~20,000	949	32.5 (29.8, 35.2)		
	≥20,000	1331	36.3 (33.6, 39.1)		
	Deficiency	115	32.5 (24.8, 40.2)		
Sleeping time (h)	<7 h	956	31.2 (28.4, 33.9)	0.534	0.766
	7~9 h	2361	32.3 (30.7, 34.0)		
	≥9 h	1049	31.7 (29.0, 34.3)		
Sedentary behavior (h)	0~2 h	783	33.3 (30.4, 36.2)	1.162	0.560
	2~4 h	1262	31.9 (29.6, 34.1)		
	≥4 h	2321	31.5 (29.8, 33.1)		
Dietary pattern	Traditional southern dietary pattern	1367	31.3 (29.4, 33.2)	1.228	0.746
	Diversified dietary pattern	1168	32.5 (30.3, 34.7)		
	Heavy oil and salt pattern	1087	32.8 (29.7, 35.8)		
	Animal oil–Other animal meat–Coarse grain pattern	744	31.1 (28.0, 34.2)		
Total		4366	31.9 (30.6, 33.2)		

**Table 5:** Association between different dietary patterns and hypertensive dyslipidemia multimorbidity.

Dietary Pattern	Group of Quartile	No. of Cases	Model 1	Model 2	Model 3
			0R (95% CI)	0R (95% CI)	0R (95% CI)
Traditional southern dietary pattern	Q1	3452	Ref	Ref	Ref
	Q2	3453	1.005 (0.840, 1.203)	1.008 (0.845, 1.204)	1.057 (0.881, 1.268)
	Q3	3452	1.036 (0.870, 1.233)	1.035 (0.868, 1.234)	1.085 (0.909, 1.296)
	Q4	3452	1.086 (0.982, 1.202)	1.085 (0.980, 1.201)	1.072 (0.963, 1.194)
Diversified dietary pattern	Q1	3452	Ref	Ref	Ref
	Q2	3453	1.079 (0.936, 1.243)	1.082 (0.939, 1.247)	1.018 (0.880, 1.177)
	Q3	3452	1.074 (0.912, 1.266)	1.074 (0.912, 1.266)	0.982 (0.828, 1.165)
	Q4	3452	1.123 (0.969, 1.302)	1.122 (0.968, 1.300)	0.999 (0.851, 1.173)
Heavy oil and salt pattern	Q1	3452	Ref	Ref	Ref
	Q2	3453	1.004 (0.903, 1.116)	1.042 (0.904, 1.202)	1.048 (0.902, 1.218)
	Q3	3452	0.985 (0.886, 1.097)	1.146 (0.977, 1.343)	1.043 (0.904, 1.204)
	Q4	3452	1.071 (0.963, 1.192)	1.055 (0.908, 1.225)	1.153 (0.982, 1354)
Animal oil–Other animal meat–Coarse grain pattern	Q1	3452	Ref	Ref	Ref
	Q2	3453	1.064 (0.961, 1.178)	1.047 (0.942, 1.164)	1.070 (0.996, 1.186)
	Q3	3452	1.061 (0.958, 1.175)	1.068 (0.960, 1.187)	1.064 (0.961, 1.179)
	Q4	3452	1.080 (0.975, 1.195)	1.095 (0.985, 1.218)	1.179 (1.032, 1.316)

Model 1 did not adjust, Model 2 adjusted age and sex on the basis of Model 1, and Model 3 adjusted BMI, physical activity level, education level, smoking status, marital status, per capita household income, alcohol consumption, sleep duration, sedentary time, regional distribution, and family history of hypertension on the basis of Model 2.

**Table 6:** Association between different dietary patterns and hypertensive dyslipidemia multimorbidity by logistic regression.

Regional Distribution	Dietary Pattern	Group of Quartile	No. of Cases	Model 1	Model 2	Model 3
				0R (95% CI)	0R (95% CI)	0R (95% CI)
South China	Traditional southern dietary pattern	Q1	107	Ref	Ref	Ref
		Q2	260	1.415 (0.700, 2.861)	1.480 (0.747, 2.935)	1.444 (0.758, 2.749)
		Q3	368	1.370 (0.528, 3.552)	1.370 (0.548, 3.424)	1.278 (0.531, 3.075)
		Q4	416	1.781 (0.706, 4.490)	1.818 (0.800, 4.132)	2.080 (1.036, 4.175)
East China	Traditional southern dietary pattern	Q1	377	Ref	Ref	Ref
		Q2	447	1.373 (0.785, 2.400)	1.405 (0.798, 2.472)	1.487 (0.858, 2.577)
		Q3	419	1.347 (0.743, 2.441)	1.369 (0.736, 2.545)	1.452 (0.801, 2.631)
		Q4	413	1.619 (0.982, 2.669)	1.670 (0.991, 2.812)	1.734 (1.052, 2.859)
	Diversified dietary pattern	Q1	174	Ref	Ref	Ref
		Q2	237	0.878 (0.515, 1.497)	0.865 (0.506, 1.478)	0.854 (0.486, 1.499)
		Q3	271	0.727 (0.407, 1.297)	0.698 (0.387, 1.261)	0.654 (0.364, 1.174)
		Q4	296	1.045 (0.615, 1.775)	1.047 (0.613, 1.787)	1.031 (0.589, 1.805)
Southwest China	Animal oil–Other animal meat–Coarse grain pattern	Q1	154	Ref	Ref	Ref
		Q2	181	1.265 (0.585, 2.737)	1.256 (0.614, 2.567)	1.496 (0.749, 2.988)
		Q3	230	1.353 (0.598, 3.059)	1.352 (0.613, 2.980)	1.953 (0.777, 4.908)
		Q4	228	1.641 (0.725, 3.712)	1.611 (0.729, 3.562)	2.009 (1.004, 4.021)
	Traditional southern dietary pattern	Q1	275	Ref	Ref	Ref
		Q2	173	1.789 (1.036, 3.091)	1.651 (0.957, 2.848)	1.675 (0.922, 3.043)
		Q3	158	0.755 (0.415, 1.374)	0.756 (0.424, 1.347)	0.741 (0.368, 1.492)
		Q4	137	0.626 (0.344, 1.140)	0.625 (0.341, 1.144)	0.686 (0.334, 1.408)
Central China	Heavy oil and salt pattern	Q1	153	Ref	Ref	Ref
		Q2	182	1.466 (0.779, 2.758)	1.524 (0.799, 2.906)	1.451 (0.710, 2.966)
		Q3	194	1.532 (0.639, 3.675)	1.556 (0.648, 3.734)	1.719 (0.819, 3.606)
		Q4	190	2.381 (1.105, 5.128)	2.401 (1.142, 5.044)	2.343 (1.107, 4.955)
	Traditional southern dietary pattern	Q1	118	Ref	Ref	Ref
		Q2	128	0.816 (0.465, 1.433)	0.832 (0.484, 1.428)	0.827 (0.488, 1.402)
		Q3	103	0.785 (0.418, 1.474)	0.793 (0.446, 1.410)	0.765 (0.440, 1.331)
		Q4	93	0.938 (0.591, 1.488)	0.946 (0.585, 1.528)	0.864 (0.477, 1.562)
North China	Diversified dietary pattern	Q1	241	Ref	Ref	Ref
		Q2	269	1.154 (0.632, 2.105)	1.108 (0.607, 2.023)	1.006 (0.560, 1.808)
		Q3	198	1.576 (1.051, 2.363)	1.596 (1.033, 2.466)	1.432 (0.802, 2.558)
		Q4	206	1.304 (0.832, 2.045)	1.316 (0.808, 2.144)	1.407 (0.706, 2.802)
	Heavy oil and salt pattern	Q1	171	Ref	Ref	Ref
		Q2	128	1.074 (0.590, 1.957)	1.073 (0.594, 1.938)	1.130 (0.573, 2.230)
		Q3	115	2.478 (1.513, 4.058)	2.432 (1.488, 3.973)	2.110 (1.198, 3.714)
		Q4	79	2.024 (1.140, 3.595)	2.009 (1.087, 3.714)	1.837 (1.019, 3.312)
Northwest China	Heavy oil and salt pattern	Q1	190	Ref	Ref	Ref
		Q2	106	1.477 (0.552, 3.947)	1.466 (0.542, 3.962)	1.352 (0.531, 3.441)
		Q3	87	0.573 (0.165, 1.994)	0.555 (0.154, 2.007)	0.476 (0.156, 1.449)
		Q4	62	0.599 (0.201, 1.790)	0.569 (0.187, 1.729)	0.624 (0.143, 2.728)
	Diversified dietary pattern	Q1	232	Ref	Ref	Ref
		Q2	103	1.332 (0.741, 2.392)	1.306 (0.732, 2.329)	1.106 (0.584, 2.093)
		Q3	62	1.596 (0.604, 4.222)	1.623 (0.572, 4.601)	1.294 (0.312, 5.363)
		Q4	29	0.483 (0.249, 0.939)	0.495 (0.251, 0.976)	0.377 (0.173, 0.824)
Northeast China	Diversified dietary pattern	Q1	116	Ref	Ref	Ref
		Q2	127	3.385 (0.653, 17.561)	3.503 (0.714, 17.194)	2.952 (0.876, 9.951)
		Q3	147	0.996 (0.364, 2.726)	0.924 (0.331, 2.579)	0.606 (0.284, 1.289)
		Q4	118	1.559 (0.719, 3.382)	1.390 (0.611, 3.162)	1.197 (0.520, 2.760)

Model 1 did not adjust, Model 2 adjusted age and sex on the basis of Model 1, and Model 3 adjusted BMI, physical activity level, education level, smoking status, marital status, per capita household income, alcohol consumption, sleep duration, sedentary time, and family history of hypertension on the basis of Model 2.

## Data Availability

The data are not allowed to be disclosed according to the National Institute for Nutrition and Health, Chinese Center for Disease Control and Prevention.

## Supplementary Materials

The following supporting information can be downloaded at: https://www.mdpi.com/article/10.3390/nu17050852/s1, Table S1: Proportion of dietary patterns among the elderly in different regions.
