# Supplementary material for: Regional Dietary Pattern Associated with the Risk of Hypertensive Dyslipidemia Multimorbidity in Chinese Elderly: Results from China Nutrition and Health Surveillance in 2015–2017
Source: Nutrients. 2025 Feb 28;17(5):852. doi: 10.3390/nu17050852 (PMC11902145; doi:10.3390/nu17050852)
Supplement: Supplementary file 1 [file nutrients-17-00852-s001.zip › nutrients-3365562-supplementary.pdf]

Supplementary materials

Table S1: Proportion of dietary patterns among the elderly in different regions

region	traditional southern dietary pattern		diversified dietary pattern		heavy oil and salt pattern		Animal oil-other animal meat-Coarse grain pattern		χ^2	*p*-Value
	frequency	proportion%	frequency	proportion%	frequency	proportion%	frequency	proportion%		
South China	1151	75.9	152	7.9	153	7.2	120	9.0	251.219	<0.001
East China	1656	35.8	1140	28.8	978	23.7	447	11.7	34.795	<0.001
Southwest China	743	37.9	157	6.4	385	17.3	793	38.4	57.376	<0.001
Central China	442	25.9	372	21.1	719	36.8	305	16.2	11.789	0.008
North China	53	3.9	914	47.7	493	26.3	348	22.1	66.984	<0.001
Northwest China	14	1.0	426	37.9	445	44.9	168	16.2	65.878	<0.001
Northeast region	237	15.3	508	46.9	275	20.9	215	16.9	44.990	<0.001
